# Analysis of risk factors associated with acute respiratory infections among under-five children in Uganda

**DOI:** 10.1186/s12889-022-13532-y

**Published:** 2022-06-17

**Authors:** Yassin Nshimiyimana, Yingchun Zhou

**Affiliations:** 1 School of Statistics, East China Normal University, Shanghai, China; 2 KLATASDS-MOE, School of Statistics, East China Normal University, Shanghai, China

**Keywords:** Acute respiratory infections, Risk-factors, Under-five mortality, Uganda

## Abstract

**Background:**

Globally, infectious diseases are the major cause of death in children under the age of 5 years. Sub-Saharan Africa and South Asia account for 95% of global child mortalities every year, where acute respiratory infections (ARI) remain the leading cause of child morbidity and mortality. The aim of this study is to analyze the risk factors of ARI disease symptoms among children under the age of 5 years in Uganda.

**Methods:**

A cross-sectional design was used to analyze 2016 Uganda Demographic and Health Survey (UDHS) data collected on 13,493 children under the age of 5 years in Uganda. Various methods, such as logistic regression, elastic net logistic regression, decision tree, and random forest, were compared and used to predict 75% of the symptom outcomes of ARI disease. Well-performing methods were used to determine potential risk factors for ARI disease symptoms among children under the age of 5 years.

**Results:**

In Uganda, about 40.3% of children were reported to have ARI disease symptoms in the 2 weeks preceding the survey. Children under the age of 24 months were found to have a high prevalence of ARI disease symptoms. By considering 75% of the sample, the random forest was found to be a well-performing method (accuracy = 88.7%; AUC = 0.951) compared to the logistic regression method (accuracy = 62.0%; AUC = 0.638) and other methods in predicting childhood ARI symptoms. In addition, one-year old children (OR: 1.27; 95% CI: 1.12–1.44), children whose mothers were teenagers (OR: 1.28; 95% CI: 1.06–1.53), and farm workers (1.25; 95% CI: 1.11–1.42) were most likely to have ARI disease symptoms than other categories. Furthermore, children aged 48–59 months (OR: 0.69; 95% CI: 0.60–0.80), breastfed children (OR: 0.83; 95% CI: 0.76–0.92), usage of charcoal in cooking (OR: 0.77; 95% CI: 0.69–0.87), and the rainy season effect (OR: 0.66; 95% CI: 0.61–0.72) showed a low risk of developing ARI disease symptoms among children under the age of 5 years in Uganda.

**Conclusion:**

Policy-makers and health stakeholders should initiate target-oriented approaches to address the problem regarding poor children’s healthcare, improper environmental conditions, and childcare facilities. For the sake of early child care, the government should promote child breastfeeding and maternal education.

**Supplementary Information:**

The online version contains supplementary material available at 10.1186/s12889-022-13532-y.

## Background

Globally, infant and child mortality rates are critical issues and fundamental indicators of a country’s population’s health, quality of life, and socioeconomic situation [1]. A remarkable decline of 60% in under-five mortality has been observed over the last three decades. However, 7.4 million annual global mortalities are estimated due to preventable and treatable diseases in young children. Besides, 70% of these deaths occur in children under the age of 5 years, and 95% are from South Asia and sub-Saharan Africa, i.e., on average, about 1 in 13 children in sub-Saharan Africa die before the age of five [2]. Various factors may contribute to high mortality rates, such as poor living conditions and other socio-economic factors of countries’ populations where childhood acute respiratory infections (ARI) remain among the top leading morbidities in low-income countries, particularly in sub-Saharan Africa [3].

The ARI disease and its related symptoms are typically caused by contagious viruses and bacterial infections that spread rapidly through droplets from either person-to-person or contaminated food or drinking water due to poor hygiene [4]. According to WHO 2019, ARI diseases are the fourth most common childhood disease among those with a higher rate of morbidity. When combined with malaria, ARI diseases become the top communicable diseases causing more deaths than other comorbidities [5–7]. In addition, the symptoms of ARI disease coincide with those of diarrhea and malaria diseases could lead to childhood death [8].

In Uganda, ARI has remained the leading cause of morbidity and mortality in children under the age of 5 years, accounting for about 9% of the ARI prevalence, with 81.3% in urban areas. The under-five mortality rate accounted for 1 in 16 child deaths, and 42% of these deaths occurred in the neonatal period [9]. The heavy loss of young lives from childhood ARI mortalities poses a heavy burden to families and healthcare providers in Uganda. Therefore, conducting research regarding the assessment of risk factors related to such diseases can greatly help policy decision-making and reduce these morbidity and mortality rates, especially in under-five children.

Traditional analysis methods such as logistic regression and chi-square test approaches are commonly applied in social science and medical literature. However, in diagnosing cardiopulmonary diseases using medical data, machine learning tools have become popular and frequently used in recent research [10]. The appropriate usage of machine learning algorithms has revealed significant performance in the prediction and classification of disease outcomes [11]. This study aims to determine potential risk factors contributing to ARI disease symptoms in children under the age of 5 years in Uganda using well-performed methods to predict the ARI symptom outcomes between traditional and machine learning analysis methods. The study findings could help in making research-based decisions to address the associated risk factors of ARI disease symptoms relevant to the disease’s control and spread among children.

## Methods

### Data source

This study used secondary data from the recent five-year cross-sectional survey, the Uganda Demographic and Health Survey (UDHS), that was conducted between June and December 2016. The UDHS is conducted by the Ugandan Bureau of Statistics and collaborated with the DHS program to collect up-to-date data for fundamental demographic and health indicators relevant to policymakers and program managers in order to evaluate the national population’s health and nutritional programs [9]. The DHS data collected in different developing countries can be found and downloaded via the website of the DHS program after approval.

### Design and sampling

We used a cross-sectional study design in collecting characteristics and information regarding the prevalence of the ARI disease symptoms among children under the age of 5 years in Uganda, using UDHS data collected in 2016. We used a two-stage stratified sampling design to select the sample. The first stage involved selecting 697 geographic areas named enumerated areas (EAs) (535 rural and 162 urban EAs) that covered 130 households on average, and the second stage involved the selection of households to be included in each EA. All the EAs with more than 300 households were segmented into one EA, and the households in the EAs were selected with a probability proportional to the size of the segment [9].

### Population and sample

The target population of this study was comprised of male and female children under the age of 5 years from different regions of Uganda. The data and recorded information for 13,493 children were used as the sample for this study. The total sample of children was divided into two groups: 75% for analysis and 25% for testing the performance of the various methods of analysis used in the study.

### Variables of interest

**Fig. 1:**
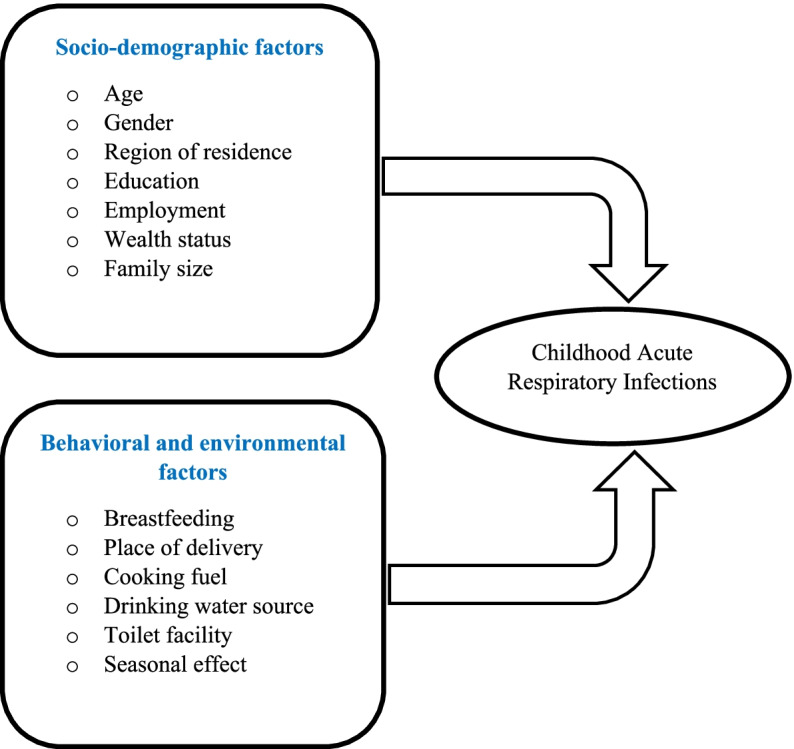
A framework of factors of childhood ARI disease symptoms

In this study, we used various characteristics that were measured in the 2016 UDHS [9] and factors from other related literature, which were included in the survey dataset (Fig. 1). Behavioral, environmental, and social demographic characteristics for children, mothers, and households were used to analyze and determine potential risk factors associated with the symptoms of ARI disease in children under the age of five in Uganda. During the survey, mothers aged 15–49 years who had children under the age of 5 years in the selected households were asked whether their children experienced ARI disease symptoms such as coughing accompanied by short, rapid or difficulty breathing in the 2 weeks before the survey. The responses regarding the ARI disease symptoms were considered subjective since they were mothers’ perceptions without validation from medical personnel. The explanations for the variables used in this study are presented in the supplementary file in Tables A1 and A2.

### Analysis methods

The scope of this study focuses primarily on determining the potential risk factors of ARI disease symptoms based on the well-performing methods between traditional and machine learning methods of analysis mostly applied in the social sciences and medical research [12].

#### Logistic regression

We used a binary logistic regression (LR) model shown in Eq. 1 to analyze the log-linear association of ***k*** variables (i.e., factors), **X**_**1**_**,X**_**2**_**,** …**,X**_**k**_ with their corresponding ***b***_**1**_***,b***_**2**_***,*** …***,b***_***k***_ effects on **Y** outcome of the ARI disease symptoms, 1 if “child had ARI symptoms” and 0 “otherwise” where ***π*** indicates the probability that a child had the ARI symptoms [13]. Stepwise variable selection procedures were also used to select influential factors associated with the outcome of interest, i.e., the symptoms of ARI disease.

1 $$\mathbf{Y}=\mathbf{\ln}\left(\frac{\boldsymbol{\pi}}{\mathbf{1}-\boldsymbol{\pi}}\right)={\boldsymbol{b}}_{\mathbf{0}}+{\boldsymbol{b}}_{\mathbf{1}}{\boldsymbol{X}}_{\mathbf{1}}+{\boldsymbol{b}}_{\mathbf{2}}{\boldsymbol{X}}_{\mathbf{2}}+\dots +{\boldsymbol{b}}_{\boldsymbol{k}}{\boldsymbol{X}}_{\boldsymbol{k}}$$

#### Elastic net regression

The elastic net logistic regression (EN) model shown in Eq. 2 was used in addition to the previous LR model in Eq. 1 to control the correlation between features in order to solve the problem of overfitting that could exist in the analysis of risk factors associated with the outcomes of the ARI disease symptoms [14]. The EN method penalizes and shrinks ***b***_**1**_***,b***_**2**_***,*** …***,b***_***k***_ effects of the non-informative **x**_**1**_**,x**_**2**_**,** …**,x**_**k**_ variables using non-negative tuning parameters ***αϵ***[**0**, **1**] and ***λ*** with ten-fold cross-validation [2].

2 $$\left(\widehat{{\mathrm b}_0,\mathrm b}\right)=\arg\;\min\;\left\{-{\textstyle\sum_{\mathrm i=1}^{\mathrm n}}\left[{\mathrm y}_{\mathrm i}\left({\mathrm b}_0+\mathrm x_{\mathrm i}^{\mathrm T}\mathrm b\right)-\mathrm{In}\left(1+\exp\left\{{\mathrm b}_0+\mathrm x_{\mathrm i}^{\mathrm T}\mathrm b\right\}\right)\right]\;+\mathrm\lambda\left[\frac12\left(1-\mathrm\alpha\right){\textstyle\sum_{\mathrm j=1}^{\mathrm k}}\mathrm b_{\mathrm j}^2+\mathrm\alpha{\textstyle\sum_{\mathrm j=1}^{\mathrm k}}{\textstyle\left|{\mathrm b}_{\mathrm j}\right|}\right]\right\}$$

#### Machine learning methods

In addition, machine learning algorithms such as decision tree (DT) and random forest (RF) methods were also used in comparisons with the regression methods to predict the outcomes of ARI disease symptoms in children under the age of 5 years in Uganda [15, 16]. The DT algorithm was particularly used due to its advantages like its tree-like structure, which is simple and easy to learn and interpret, while the RF algorithm approach was used as an extension of the DT method because of its effectiveness in minimizing the variance using its random DT tree-like structures generated from a random sample in the prediction [17].

### Measures of evaluation

In the evaluation of the performance of the methods used in this study, we considered various measures or metrics that are applied in the contingency matrix in diagnosing ill patients in most medical research [18]. A *total accuracy* in Eq. 3 measures the proportion of all children reported as with and without ARI disease symptoms who are correctly predicted by the method in this study; a *precision* measure in Eq. 4 shows the proportion of children who actually had ARI symptoms and were correctly predicted as having ARI disease symptoms. While the *selectivity* measure shown in Eq. 5 measured the proportion of children who were actually reported as not having ARI symptoms and correctly predicted by the method as not having ARI symptoms; A *recall* measure in Eq. 6, also called a sensitivity measure, indicates the proportion of the children who are predicted as symptomatic among all children with ARI symptoms in the study. We also used the *area under the curves* (AUCs) measure for the receiver operating characteristic (ROC) curves based on the true and predicted outcomes of ARI symptoms [10]. This study used statistical software such as STATA version 17.0 for data management and R software using functions in the Caret package for analyzing data.

3 $$\mathrm{Accuracy}=\frac{\left( TP+ TN\right)}{\left( TP+ FP\right)+\left( TN+ FN\right)}$$

4 $$\mathrm{Precision}=\frac{TP}{\left( TP+ FP\right)}$$

5 $$\mathrm{Selectivity}=\frac{TN}{\left( TN+ FP\right)}$$

6 $$\mathrm{Recall}\ \mathrm{or}\ \mathrm{Sensitivity}=\frac{TP}{\left( TP+ FN\right)}$$

Where TP, TN, FP, and FN represent the number of true positives, true negatives, false positives, and false negatives respectively.

## Results

**Fig. 2:**
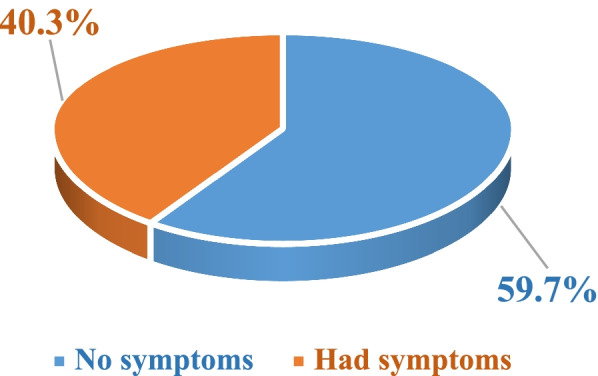
Prevalence of ARI disease symptoms in children under the age of 5 years in Uganda

In this study, a sample of 13,493 children under the age of 5 years in Uganda was analyzed. Overall, the prevalence of ARI disease symptoms in children with symptoms was found to be 5437 (40.3%) and 8056 (59.7%) for children without ARI disease symptoms (Fig. 2). Tables 1 and 2 show that the symptoms’ prevalence of ARI disease in children was high in males (50.7%) compared to females (49.3%), and about 44.5% of children with ARI symptoms were under 24 months of age, and 33.8% had mothers under 25 years of age and living in a lower-income class (47.4%). About 74.2% of children had mothers who only attended below the secondary level of education, and only 56.6% were breastfed. The majority of children reported were found in households exposed to wood smoke from firewood as cooking energy (77.9%) and 53.4% reported in the dry season.

**Table 1:** Distribution of the ARI symptoms’ prevalence based on socio-economic and demographic characteristics

Characteristics	ARI disease symptoms: ***n*** = 13,493			Sig.
	No (%)	Yes (%)	Total (%)	
Child age (months)				
0–11	1724 (21.4)	1149 (21.1)	2873 (21.3)	< 0.001
12–23	1440 (17.9)	1273 (23.4)	2713 (20.1)	
24–35	1534 (19.0)	1124 (20.7)	2658 (19.7)	
36–47	1608 (20.0)	1008 (18.5)	2616 (19.4)	
48–59	1750 (21.7)	883 (16.3)	2633 (19.5)	
Child gender				
Male	3991 (49.5)	2757 (50.7)	6748 (50.0)	0.183
Female	4065 (50.5)	2680 (49.3)	6745 (50.0)	
Region of residence				
Central	1358 (16.9)	1360 (25.0)	2718 (20.1)	< 0.001
Eastern	2346 (29.1)	1517 (27.9)	3863 (28.6)	
Northern	2023 (25.1)	1357 (25.0)	3.380 (25.1)	
Western	2329 (28.9)	1203 (22.7)	3532 (26.2)	
Mother age (years)				
15–19	441 (5.5)	364 (6.7)	805 (6.0)	< 0.001
20–24	2165 (26.9)	1474 (27.1)	3639 (27.0)	
25–29	1991 (24.7)	1466 (27.0)	3457 (25.6)	
30–34	1716 (21.3)	1072 (19.7)	2788 (20.7)	
35–39	1094 (13.6)	670 (12.3)	1764 (13.1)	
40–49	649 (8.1)	391 (7.2)	1040 (7.7)	
Mother education				
No education	1097 (13.6)	705 (13.0)	1802 (13.4)	0.002
Primary	5110 (63.4)	3328 (61.2)	8438 (62.5)	
Secondary	1449 (18.0)	1088 (20.0)	2537 (18.8)	
Tertiary	400 (5.0)	316 (5.8)	716 (5.3)	
Mother employment				
Unemployed	1481 (18.4)	698 (12.8)	2179 (16.1)	< 0.001
Farmer	3947 (49.0)	2380 (43.8)	6327 (46.9)	
Other	2628 (32.6)	2359 (43.4)	4987 (37.0)	
Mother wealth status				
Lower	4012 (49.8)	2579 (47.4)	6591 (48.8)	0.004
Middle	1557 (19.3)	1033 (19.0)	2590 (19.2)	
Higher	2487 (30.9)	1825 (33.6)	4312 (32.0)	

Sig. 5% Chi-square test significance

**Table 2:** Distribution of the ARI symptoms’ prevalence based on behavioral and environmental characteristics

Characteristics	ARI disease symptoms: ***n*** = 13,493			Sig.
	No (%)	Yes (%)	Total (%)	
Family size				
Not crowded (≤ 5)	3746 (46.5)	2581 (47.5)	6327 (46.9)	0.267
Crowded (> 5)	4310 (53.5)	2856 (52.5)	7166 (53.1)	
Breastfeeding				
Not breastfed	3323 (41.3)	2362 (43.4)	5685 (42.1)	0.011
Breastfed	4733 (58.7)	3075 (56.6)	7808 (57.9)	
Child received IP drug				
No	3615 (44.9)	2363 (43.5)	5978 (44.3)	0.105
Yes	4441 (55.1)	3074 (56.5)	7515 (55.7)	
Place of delivery				
Home	2257 (28.0)	1383 (25.4)	3640 (27.0)	0.005
Public Hospital	1464 (18.2)	1030 (18.9)	2494 (18.5)	
Health Center	3225 (40.0)	2205 (40.6)	5430 (40.2)	
Private Hospital	1110 (13.8)	819 (15.1)	1929 (14.3)	
Toilet facility				
With slab	2320 (28.8)	1645 (30.3)	3965(29.4)	0.122
Without slab	4893 (60.7)	3208 (59.0)	8101(60.0)	
No facility	843 (10.5)	584 (10.7)	1427 (10.6)	
Cooking fuel				
Wood	6370 (79.1)	4237 (77.9)	10,607 (78.6)	0.112
Charcoal	1686 (20.9)	1200 (22.1)	2886 (21.4)	
Drinking water source				
Protected	6097 (75.7)	4179 (76.9)	10,276 (76.2)	0.115
Unprotected	1959 (24.3)	1258 (23.1)	3217 (23.8)	
Season effect				
Dry	3436 (42.6)	2905 (53.4)	6341 (47.0)	< 0.001
Rainy	4620 (57.4)	2532 (46.6)	7152 (53.0)	

*IP* Intestinal Parasites

### Comparison of method performances

**Fig. 3:**
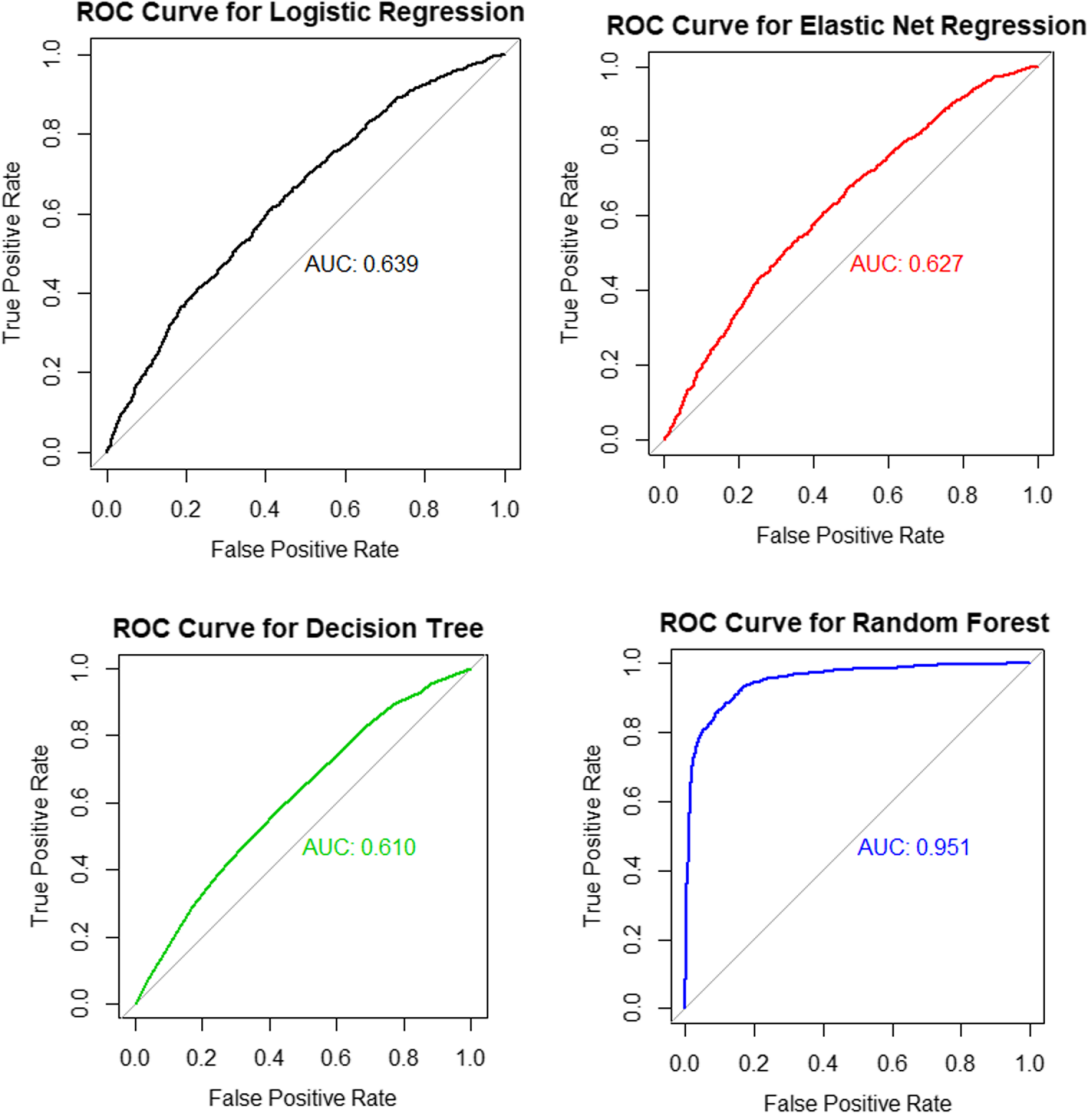
ROC curves and AUC values for method performances

The scope of this study focused primarily on determining the potential risk factors of ARI disease symptoms based on well-performing methods. In the analysis, we used 75% of the total sample as a training sample and the remaining 25% for testing the method’s performance using ten-fold cross-validation. Table 3 shows the results of the performance comparisons between logistic regression (LR), elastic net logistic regression (EN), decision tree (DT), and random forest (RF) methods. The RF method showed the highest accuracy of 88.7 and 93.10% for precision in predicting the childhood ARI symptoms compared to other methods, i.e., about 88.7% of children who actually reported having or not having symptoms of ARI were correctly predicted by the RF method, while 93.1% of children with actual symptoms were also correctly predicted to have ARI symptoms. The LR method is followed with 62.0% accuracy and 88.03% precision. The other methods, such as the EN methods (61.7% accurate and 86.25% precise) and the DT method (61.2% accurate and 83.0% precise), showed the least performance in the prediction of ARI disease symptoms in this study. The AUC results for the receiver operating curves comparing these methods are presented in Fig. 3. Therefore, we used the random forest and logistic regression methods to determine potential risk factors for ARI disease symptoms among children under the age of 5 years in Uganda.

**Table 3:** Comparison of predictive performances for the methods

Method	Recall	Selectivity	Precision	Accuracy	AUC
Logistic regression (LR)	**63.05%**	**57.04%**	**88.03%**	**62.05%**	**0.638**
Elastic net regression (EN)	63.14%	55.47%	86.25%	61.73%	0.627
Decision tree (DT)	63.34%	53.33%	82.97%	61.16%	0.610
Random forest (RF)	**88.57%**	**88.93%**	**93.10%**	**88.70%**	**0.951**

*AUC* area under the curve

### Potential risk factors contributing to the childhood ARI disease symptoms

Tables 4 and 5 summarize the logistic regression and random forest methods’ results. A subsample of 75% of all sampled children used in performance comparisons between these methods was used to determine the potential risk factors for ARI disease symptoms among children in Uganda. Adjusting for other factors, the LR model results (Table 4) reveal that children aged 12–23 months had a higher risk of 1.27 times (95% CI: 1.12–1.44) of developing ARI disease symptoms than infants, while children aged 48–59 months showed a low risk of 0.69 times (95% CI: 0.60–0.80) of ARI symptoms compared to children at earlier ages. Children living in other regions different from the central region had a low risk of developing ARI symptoms. Children of teen mothers had a significantly higher risk of having ARI symptoms than children of mothers in their middle ages (21–24 years old), and employed mothers in farming 1.25 times (95% CI: 1.11–1.42) or other employment 1.93 times (95% CI: 1.71–2.19) showed that their children were associated with a high risk of ARI disease symptoms compared to children of unemployed mothers who had enough time to care for them.

**Table 4:** Logistic regression estimates of risk factors associated with childhood ARI symptoms in Uganda

Factors	Frequency (%)	AOR	[95% CI]	Sig.
**Socio-demographic factors**				
Child age (months)				
0–11	2131 (21.1)	Ref	–	–
12–23	2070 (20.4)	1.27	[1.12–1.44]	< 0.001
24–35	2003 (19.8)	0.98	[0.85–1.12]	0.727
36–47	1939 (19.2)	0.88	[0.76–1.01]	0.065
48–59	1977 (19.5)	0.69	[0.60–0.80]	< 0.001
Region of residence				
Central	2064 (20.4)	Ref	–	–
Eastern	2877 (28.4)	0.63	[0.56–0.72]	< 0.001
Northern	2535 (25.1)	0.65	[0.57–0.74]	< 0.001
Western	2644 (26.1)	0.51	[0.44–0.57]	< 0.001
Mother age (years)				
15–19	595 (5.9)	1.28	[1.06–1.53]	0.009
20–24	2736 (27.0)	Ref	–	–
25–29	2586 (25.6)	1.07	[0.95–1.19]	0.268
30–34	2067 (20.4)	0.94	[0.83–1.06]	0.313
35–39	1334 (13.2)	0.96	[0.84–1.11]	0.600
40–49	802 (7.9)	0.95	[0.80–1.12]	0.546
Mother employment				
Unemployed	1624 (16.1)	Ref	–	–
Farmer	4780 (47.2)	1.25	[1.11–1.42]	< 0.001
Other	3716 (36.7)	1.93	[1.71–2.19]	< 0.001
**Behavioral and environmental factors**				
Breastfeeding				
Not Breastfed	4247 (42.0)	Ref	–	–
Breastfed	5873 (58.0)	0.83	[0.76–0.92]	< 0.001
Cooking energy				
Wood	7955 (78.6)	Ref	–	–
Charcoal	2165 (21.4)	0.77	[0.69–0.87]	< 0.001
Season effect				
Dry	4769 (47.1)	Ref	–	–
Rainy	5351 (52.9)	0.66	[0.61–0.72]	< 0.001

Sig.: *p*-value at 5% level of significance

**Table 5:** Potential risk factors contributing to the childhood ARI disease in both random forest and logistic regression methods

	Random Forest (RF)	Logistic Regression (LR)
	Mother employment^a^	Region of residence
	Season effect^a^	Mother employment
	Region of residence^a^	Season effect
	Cooking energy^a^	Child age
	Mother wealth status	Cooking energy
	Place of delivery	Breastfeeding
	Mother education	Mother age
**Accuracy**	**88.70%**	**62.05%**

^a^indicates factors in both methods

In behavioral and environmental factors, mothers who breastfed their children had a lower risk of 0.83 times (95% CI: 0.76–0.92) of ARI symptoms compared to those who did not breastfeed, and other factors such as the child’s exposure to charcoal cooking smoke showed a lower risk of 0.77 times (95% CI: 0.69–0.87) of developing ARI disease symptoms than children exposed to firewood smoke, while in the rainy season, children were less likely to 0.66 times (95% CI: 0.61–0.72) of developing symptoms of ARI disease than in the dry season in Uganda.

For the random forest method, the important factors contributing to the prediction of ARI disease symptoms were shown in Table 5, and risk factors such as mother’s employment, season effect, region of residence, cooking energy, mother’s wealth status, place of delivery, and mother’s education were potential risk factors contributing to the ARI disease symptoms among children under the age of 5 years in the random forest methods. 

## Discussion

This study builds upon the analysis of risk factors for ARI disease symptoms among children under the age of 5 years and compares various methods’ performances in predicting childhood ARI symptom outcomes. Using well-performing methods, we analyzed socio-demographic, behavioral, and environmental factors contributing to childhood ARI disease symptoms in Uganda using the 2016 UDHS dataset. The results revealed that the random forest method performed better in accuracy than other methods considered in the analysis, followed by the logistic regression method (Table 5). As shown in two methods, the employment of mothers in farming activities, the season effect, the region of residence, and the fuel used for cooking, such as firewood and charcoal, were found to be potential risk factors contributing to the childhood ARI disease symptoms in Uganda. In addition, the young ages of mothers and children, breastfeeding, and wealth status were also found to be factors associated with ARI disease symptoms among children in this study.

Other studies conducted in Uganda also showed that these higher prevalence results for childhood ARI diseases were consistent with the current findings [19, 20], and the high risk of childhood ARI disease symptoms due to factors such as season and geographical regions was also in concurrence with other findings from studies conducted in neighboring countries such as Rwanda and Kenya [21–23]. A vulnerable region in Uganda, like the northern region where people were forced to settle in camps because of the civil war in 1986, suffered from overcrowding and poor sanitation that speeded up the disease occurrence [19]. More efforts in sanitation and appropriate health services from the government should be established in highly risk areas to eliminate regional differences against ARI diseases. However, a study conducted in the Gulu district, northern Uganda, reported that children living in urban areas were more likely to develop ARI symptoms than those living in rural areas [24].

The study findings also revealed that the ARI symptoms increased among the children exposed to firewood smoke compared to those exposed to charcoal smoke. These results of the association between wood fuel and ARI symptoms were similar to others conducted in sub-Saharan African countries [23, 25–28]. According to WHO reports, “Children exposed to cooking fuels and parental smoking are more likely to be at a high risk of having pneumonia and other respiratory infection diseases” [8]. The need for parents’ and community education about the dangers of smoking to children must be addressed, especially in places where smoking and firewood are used frequently [29].

The ARI factors, such as the education and employment of mothers, are consistent with other results found in Kenya, Ethiopia, and Rwanda [22, 30, 31]. However, the factors contradict findings from another study conducted in northern Uganda because of the discrepancies in living standards and characteristics of the population studied. In the northern part, people suffered from overcrowding and poor sanitation, and most people were living in camps that encouraged disease occurrence and easy spread [19]. In the current study, children younger than 1 year old showed a higher risk of having ARI disease symptoms than children aged 48–59 months. These findings are supported by similar findings [29, 32–34]. The factors were related to the low rates of immunization in young children, low maternal literacy, and the young mothers in farming activities that do not allow the care of young children, particularly in sub-Saharan African countries, where health facilities and maternal healthcare education have to be improved.

Aside from the foregoing, this study provides evidence on parental behavior factors such as breastfeeding, which contradicts other findings [19, 20]. The current study showed that non-breastfed children whose mothers were teenaged were found to be more likely to develop ARI disease symptoms than breastfed ones, and generally, breastfeeding is more important to the child’s nutrition and the good functionality of the child’s immunity system.

Despite the strengths, limitations also have to be discussed. Parental smoking and childbirth weight factors were found to be significantly associated with ARI disease among children under the age of five in other studies [26, 29, 35, 36]. Due to the much missing information presented in these two variables in the current study, these two risk factors were limited in the 2016 UDHS dataset. In general, smoking harms the natural human defense of the respiratory system [37], especially in low birth-weight children. The government and community campaigns should educate people about the dangers of smoking on people’s health, particularly in young children’s households.

In terms of the analysis methods, we used both new and traditional supervised analysis methods, such as machine learning algorithms and multivariate regression methods, to predict the childhood outcomes of ARI disease symptoms. Furthermore, these findings complement other comparative machine learning findings [38–41] in providing evidence of the better performance of the random forest algorithm (88.7%) than traditional methods of analysis. However, other studies [42, 43] contradicted these findings. Further research is needed to overcome these challenges and compare various analysis methods using nationwide cross-sectional survey datasets like the DHS data. Moreover, longitudinal data analysis can better examine the potential risk factors of ARI disease in children under the age of 5 years.

In summary, this paper revealed that the mother’s employment and age, child age, breastfeeding, wealth status, season effect, region of residence, and cooking fuel such as firewood and charcoal were found to be potential risk factors for ARI disease symptoms in children under the age of 5 years. In this study, non-breastfed children whose mothers were teenagers had a significant effect on the development of ARI disease symptoms. Based on the results, policy-makers and health stakeholders should initiate target-oriented approaches to address the problems regarding poor children’s healthcare, improper environmental conditions, and childcare facilities. The government and child family interventions have to encourage maternal education and especially child breastfeeding. For the sake of early child care, the government should promote child breastfeeding and maternal education.

## Supplementary Information

**Additional file 1: Table 1.** Descriptions of socio-economic and demographic characteristics. **Table 2.** Descriptions of behavioral and environmental characteristics.

## Data Availability

The data used is openly available to the DHS Program after having access approval at https://www.dhsprogram.com/data/

## Abbreviations

ARI: Acute Respiratory Infections
UDHS: Uganda Demographic and Health Survey
EA: Enumeration Area
LR: Logistic Regression
EN: Elastic Net Regression
DT: Decision Tree
RF: Random Forest
AOR: Adjusted Odds Ratio
CI: Confidence Interval
TP: True Positive
TN: True Negative
FP: False Positive
FN: False Negative
