# Supplementary material for: Analysis of risk factors associated with acute respiratory infections among under-five children in Uganda
Source: BMC Public Health. 2022 Jun 17;22:1209. doi: 10.1186/s12889-022-13532-y (PMC9205046; doi:10.1186/s12889-022-13532-y)
Supplement: Supplementary file 1 — Additional file 1: Table 1. Descriptions of socio-economic and demographic characteristics. Table 2. Descriptions of behavioral and environmental characteristics. [file 12889_2022_13532_MOESM1_ESM.docx]

1. **Brief Descriptions of Variables of Interest:**

Table 1 and Table 2 below show various characteristics used in the study analysis and measured in the 2016 UDHS survey dataset [9]. Behavioral, environmental, and social demographic characteristics for children, mothers, and households were used to analyze and determine potential risk factors associated with the symptoms of acute respiratory infections in children under the age of five in Uganda. During the survey, mothers aged 15–49 years who had children under the age of five years in the selected households were asked whether their children had experienced ARI disease symptoms such as the cough accompanied by short, rapid, or difficulty breathing in the 2 weeks before the survey, and the responses were considered the outcomes of interest in this study. The reported responses to ARI disease symptoms were subjective since they were mothers’ perceptions without any validation from medical personnel.

**Table 1. Descriptions of socio-economic and demographic characteristics**

| **Variable** | **Type** | **Description** |
| --- | --- | --- |
| **Outcome variable** |  |  |
|  |  |  |
| ARI disease symptoms | dummy | ARI responses were categorized into 0 “No” for the child who was reported not having symptoms, and 1 ”Yes” for the child who was reported having symptoms for ARI disease in two weeks preceding the survey. |
| **Socio-demographic characteristics** | | |
|  |  |  |
| Child age | categorized | Children's ages were measured in terms of months from 0 months to 59 months of birth categorized into five age groups. |
|  |  |  |
| Child gender | Nominal | Children of both gender, males and females were used, 0 for male and 1 for female. |
|  |  |  |
| Mother age | categorized | The ages for children mothers were included based on the reproductive ages (15 to 49 years old) categorized into six age groups. |
|  |  |  |
| Region of residence | Nominal | The Uganda districts were classified into four regions (central, eastern, northern, and western region) based on their locations in the country. |
|  |  |  |
| Mother education | Nominal | This factor was used based on the levels of education such as primary, secondary, higher, and considered also those who were not educated. |
|  |  |  |
| Mother employment | Nominal | Women whose occupations were based on agriculture and farming were considered as farmers while those who worked in other occupations were used as other categories, not employed for otherwise. |
|  |  |  |
| Mother wealth status | Nominal | The family income wealth background of the child was considered and grouped as were grouped as a lower class for poorer and poor families, higher class for rich and richer families, and middle class for neither poor nor rich. |

**Table 2. Descriptions of behavioral and environmental characteristics**

| **Variable** | **Type** | **Description** |
| --- | --- | --- |
|  |  |  |
| Family size | categorized | The numbers of people in the household were used as a crowding factor contributing to the ARI disease symptoms. The number of people less than 6 were considered as not crowded households, otherwise crowded households. |
|  |  |  |
| Breastfeeding | Nominal | Mothers were asked if they were breastfed their children. And was categorized into breastfed and not breastfed. |
|  |  |  |
| Child received IP drug | Nominal | Mothers were asked if the child received a drug for intestinal parasites in the last 6 months. It was a “Yes” and “No” question. Coded 1 for Yes, and 0 otherwise. |
|  |  |  |
| Place of delivery | Nominal | The various possible places of delivery for children were also used in contributing factors of childhood ARI illness where mother’s delivery sites such as private and public hospitals, centers and posts, and home places were considered. |
|  |  |  |
| Toilet facility | Nominal | Different household types of toilets were considered based on their protective likelihood. Households with slab, flush, improved pit latrines were grouped into one group while a household with other types of toilets was in another group and otherwise no facilities. |
|  |  |  |
| Cooking fuel | Nominal | Since the majority of households used unimproved materials when cooking. The popular materials were wood and charcoal that was air polluted materials were considered as risk factors of ARI illness. |
|  |  |  |
| Drinking water source | Nominal | The household with water sources was from unprotected spring, rainwater, and river or dam were considered as unprotected sources while those from piped, protect well and spring was protected sources of drinking water. |
|  |  |  |
| Season effect | Nominal | The country’s climate risk factor was also considered. Ugandan two annual season periods such as the dry season from December to February and June to August and the rainy season from September to November and March to May were measured based on the time the mothers’ were interviewed. |
